# Efficacy and Predictive Factors of Intravesical Botulinum Toxin A Injection for Treating Neurogenic Detrusor Overactivity in Children: A Single-Center Retrospective Study

**DOI:** 10.3390/toxins17040202

**Published:** 2025-04-17

**Authors:** Chun-Kai Hsu, Han-Yu Lin, Stephen Shei-Dei Yang, Wan-Ling Young, Shu-Yu Wu

**Affiliations:** 1 Department of Urology, Taipei Tzu Chi Hospital, Buddhist Tzu Chi Medical Foundation, New Taipei City 231016, Taiwan; svevi0614@gmail.com (C.-K.H.); urolyang@gmail.com (S.S.-D.Y.); lisayoung1345@gmail.com (W.-L.Y.); 2 School of Medicine, Tzu Chi University, Hualien 97004, Taiwan; bbkeric@gmail.com; 3 Department of Anesthesiology, Taipei Tzu Chi Hospital, Buddhist Tzu Chi Medical Foundation, New Taipei City 231016, Taiwan

**Keywords:** botulinum toxin, pediatric urology, neurogenic bladder, urodynamic study, lower urinary tract dysfunction

## Abstract

Neurogenic detrusor overactivity (NDO) is a complex condition associated with detrusor overactivity, reduced bladder compliance, and high intravesical pressures, potentially leading to urinary tract infections (UTIs) and renal impairment. This retrospective study evaluated the safety and potential efficacy of intravesical botulinum toxin A (BoNT/A) injections in children with NDO at a single institution. Eighteen pediatric patients (median age: 8.1 years) were followed for a median of 6.3 years. At follow-up, 77.8% achieved a global response assessment (GRA) score of ≥2. A statistically significant improvement was found in cystometric bladder capacity (*p* = 0.041), but it did not remain significant after Bonferroni correction, while other urodynamic trends were not statistically significant. Adverse events were infrequent, with only 11.8% experiencing mild febrile UTIs. While some patients with poorer baseline bladder conditions reported greater subjective improvement, no statistically significant predictors of success were identified. Overall, intravesical BoNT/A injection appears to be a safe and potentially effective option for managing pediatric NDO, though larger prospective studies are needed to confirm these findings.

## 1. Introduction

Neurogenic detrusor overactivity (NDO) is a complex disease that has a significant impact on patients’ health and daily activities. It commonly entails detrusor overactivity (DO), urinary leakage, vesicoureteral reflux (VUR), and hydronephrosis, and patients typically present with reduced bladder capacity, low bladder compliance, and abnormally high intravesical pressures. If proper treatment is not provided immediately, a urinary tract infection (UTI) or even a loss of kidney function may arise in particularly severe cases [1,2]. Pediatric patients are one of the most complex and vulnerable groups when it comes to NDO, as they have a relatively high risk of UTIs and renal impairment. Diagnosis and treatment are particularly challenging for pediatric patients and their families due to the disease’s adverse developmental and psychological effects [3].

The primary treatment for neurogenic bladder typically includes bladder management such as clean intermittent catheterization (CIC), long-term indwelling catheterization, and oral medications (e.g., anticholinergics, beta-3 agonists) [4,5]. Regardless of the treatment type, however, the primary goal is to avoid abnormally high intravesical pressures and protect renal function. However, if the bladder pressure does not decrease as expected following treatment, or if the patient has persistent lower urinary tract symptoms, botulinum toxin-A (BoNT/A) may be an appropriate next step before pursuing irreversible interventions [6]. Many studies have shown intravesical BoNT/A injections to prompt both objective and subjective improvements in NDO, with repeated injections usually being necessary to achieve satisfactory results [7,8,9,10,11].

Although several retrospective studies have explored the efficacy of BoNT/A in pediatric NDO, the ability to predict individual treatment responses remains limited. Prior studies have suggested that factors such as the patient’s age, baseline bladder compliance, detrusor pressure, and cystometric bladder capacity may influence outcomes. However, these findings have been inconsistent and often underpowered. For example, while some studies suggest that lower pre-injection bladder capacities or higher detrusor pressures may predict greater benefits, others have found no significant associations. Furthermore, the role of prior BoNT/A injections or concurrent medication use remains poorly defined in pediatric populations.

A recent systematic review and meta-analysis by Panunzio et al. [12] synthesized the available pediatric data on BoNT/A and reported an inverse correlation between the dryness rate and age. However, the findings across studies remain heterogeneous due to differences in outcome definitions, injection protocols, and patient characteristics. Dose–response relationships and treatment durability are also inconsistently reported. These limitations underscore the ongoing need for real-world data to identify reliable predictors of treatment response in pediatric populations. To date, no definitive parameters have been established to reliably predict treatment success [9,12,13,14]. While previous reviews have highlighted intravesical botulinum toxin A (BoNT/A) as a safe and less invasive alternative to major reconstructive surgery, there remains a need to better define optimal patient selection and long-term treatment efficacy [15]. In light of this, the aim of our study is to assess the efficacy of intravesical BoNT/A in the treatment of neurogenic detrusor overactivity (NDO) in children, with a particular focus on identifying potential predictors of treatment success and refining criteria for patient selection.

## 2. Results

A total of 18 children (median age: 8.1 years), including 14 boys and 4 girls, were enrolled in this study. No cases were excluded from our study due to missing essential information. Among these patients, 14 (77.8%) had NDO caused by myelomeningocele, 3 (16.7%) had it due to prior pelvic surgery, and 1 had it due to congenital encephalopathy. The most commonly reported subjective reasons for the first visits were urinary retention (72.2%), incontinence (50%), frequency (22.2%), urgency (16.7%), and nocturia (16.7%). Most of the patients relied on catheterization for voiding, with 15 (83.3%) using CIC and 1 using a urethral catheter. Only two children were able to maintain self-voiding without catheterization. Thirteen children presented with VUR, including three with high-grade VUR (grades IV and V). Additionally, ten of them had hydronephrosis at baseline.

Eight patients received a single intravesical BoNT/A injection, and ten patients received repeated injections, though only one patient received a third injection. None of the patients received more than three injections. The patients reported good outcomes, with a high proportion (77.8%) of patients having a GRA score of ≥2. Urinary incontinence and oral medication use both declined by 44.4%. The incidence of adverse events was low, with only two children in the study having reported mild febrile urinary tract infections, which were treatable with oral antibiotics. No serious adverse events occurred throughout the entire follow-up period. Table 1 summarizes the clinical details, demographics, urodynamic parameters, adverse events, and outcomes.

For the entire study population, a statistically significant improvement was observed in cystometric bladder capacity (uncorrected *p* = 0.041), but it did not remain significant after Bonferroni correction (adjusted threshold *p* < 0.017). The other urodynamic variables did not reach statistical significance under this more stringent criterion. The other measurable urodynamic parameters, such as bladder compliance and detrusor pressure, showed no significant change following intravesical BoNT/A injection. Although not statistically significant (*p* > 0.1), slight numerical improvements were observed in bladder compliance and detrusor pressure. These trends are reported descriptively and should not be overinterpreted. Table 2 presents the detailed results between the baseline and the post-therapy period. Table 3 presents the results of the comparison between single-injection and repeated-injection treatments.

When comparing the single- and repeated-injection groups, no statistically significant differences were observed in the urodynamic parameters. However, given the small subgroup sizes, this analysis is underpowered, and the results should be interpreted as exploratory. It is noteworthy that, in the repeated-injection group, bladder compliance increased, while detrusor pressure did not decrease.

When comparing the baseline clinical and urodynamic parameters between the successes and the failures (based on their GRA values), we found that children with better post-injection outcomes tended to be older, with a lower pre-injection bladder capacity and higher detrusor pressure (Table 4). Despite the apparent trend described above, the exploratory regression analyses were limited by the small sample size (N = 18) and the inclusion of four variables, which likely led to model overfitting and violated the recommended events-per-variable rule. As such, the regression findings should be interpreted with caution.

## 3. Discussion

This study suggests that intravesical BoNT/A injection may be a safe and potentially effective option for treating pediatric NDO, though the conclusions are limited by the small sample size and exploratory nature of the analyses. The bladder capacity of the entire treatment group improved following the treatment. For some patients, the therapeutic effect may not be evident after the first injection; however, similar benefits may be achieved through the provision of multiple injections. Consequently, patient satisfaction was generally high, but this observation should be interpreted cautiously given the small sample and lack of statistically significant findings supporting efficacy across all measures.

The primary goal of treatments for lower urinary tract problems should always be the preservation of renal function. Other important objectives include improving continence, preventing UTIs, and enhancing quality of life as much as possible. Bladder management through catheterization remains the cornerstone of treatment, though it is often paired with pharmacological therapies, such as anticholinergic agents and beta-3 agonists, to reduce DO while maintaining high detrusor compliance and age-appropriate bladder capacity [9,16]. Various antimuscarinics have been used worldwide for many years, but oxybutynin is the only agent that has been approved by the FDA for use in children. Its effectiveness is well established, but its adverse effects—such as dry eyes, dry skin, dry mouth, constipation, and gastrointestinal disturbance—have also been well documented [17]. Its side effects may require dose adjustments or even discontinuation should they prove to be problematic [18]. Beta-3 agonists have emerged as a solid alternative or an add-on therapy [19]. When pharmacological treatments fail, surgical interventions (e.g., bladder augmentation) may be considered as a final solution. However, such complex surgical procedures are irreversible and can lead to severe long-term complications, including ureteral stenosis, urinary tract stone formation, infections, and metabolic disturbances [20,21,22]. Therefore, a thorough evaluation should be conducted before pursuing major, irreversible surgery, especially in pediatric patients.

Accurate diagnosis and appropriate treatment are always crucial, especially in vulnerable patient populations, including children with NDO. Therefore, conducting both pre- and post-treatment urodynamic studies is mandatory for children undergoing intravesical BoNT/A injection. The commonly reported outcomes pertain to detrusor pressure, bladder capacity, and bladder compliance [10,23]. A VUDS plays a pivotal role in the assessment and management of children with NDO. This diagnostic tool integrates urodynamic measurements with fluoroscopic imaging, providing a comprehensive evaluation of bladder function, compliance, capacity, and coordination between the bladder and sphincter. Critically, a VUDS facilitates the detection of high-risk features, such as DO, poor bladder compliance, and vesicoureteral reflux, which are critical predictors of urinary tract complications and renal function deterioration [23,24]. The early identification of these risk factors enables timely interventions to preserve renal function and prevent complications such as hydronephrosis and recurrent UTIs. Additionally, a VUDS provides valuable anatomical insights that can aid medical providers in providing patients with tailored treatment plans [23,24]. Its comprehensiveness makes a VUDS an essential tool for optimizing long-term outcomes and improving quality of life among pediatric patients with NDO.

The use of BoNT/A in the field of NDO treatments has increased significantly since Schurch et al.’s first report on the matter over two decades ago [25]. Since a series of successful clinical trials, intravesical BoNT/A injection has become the standard of care for treating NDO refractory to antimuscarinics in adults with spinal cord injury and multiple sclerosis. The first report of treating children with intravesical BoNT/A injection was published by Schulte-Baukloh in 2002 [26]; since then, multiple studies have demonstrated that intravesical BoNT/A injections can improve both the urodynamic parameters and clinical symptoms of those with NDO. The clinical outcomes of this treatment have been favorable, with urinary incontinence being completely resolved in one-third to nearly 100% of patients. Additionally, a high percentage of patients have achieved clinical incontinence resolution, although the degree of improvement has varied across different studies [8,26,27,28].

In our study, we used a trigone-sparing injection technique with 20 intravesical sites and a dose of 5–10 U/kg, not exceeding 200 U. This approach is consistent with that in many pediatric studies; however, injection practices vary widely in terms of the site number, the dosage, and whether or not the trigone is included. Some investigators advocate for trigone-including injections to enhance efficacy, while others avoid the trigone to minimize the risk of vesicoureteral reflux. As no consensus has been reached on the optimal injection protocol in children, future comparative studies are needed to standardize the technique and improve its outcomes.

According to a previous systematic review, bladder compliance, detrusor pressure, and cystometric bladder capacity are the most frequently used parameters in outcome analysis [9]. This study also recorded bladder compliance, cystometric bladder capacity, and detrusor pressure as outcome measures. Ideally, a successful intravesical BoNT/A injection treatment would result in increased bladder compliance and bladder capacity, as well as decreased detrusor pressure. This study found only the cystometric bladder capacity to have improved. Although we stratified the outcomes by injection frequency in the subgroup analysis, our primary analysis aggregated all patient outcomes. This approach may obscure potential differences in treatment efficacy between single and repeated injections. Future studies with larger sample sizes are needed to explore how cumulative BoNT/A exposure influences response patterns over time. There was a trend toward improvements in bladder compliance and detrusor pressure, but these were not statistically significant, likely due to the small sample. One possible reason is that patients may respond differently to botulinum toxin, including variations in the required dose and duration of action after injection. Another potential explanation is the presence of undetected bladder outlet obstruction. However, we had already conducted a VUDS, which should help rule out this possibility to the greatest extent. This limitation may also explain why, when the participants were subgrouped by the number of injection sessions, none of the parameters demonstrated statistically significant differences. This also means, however, that if a single injection is not effective, repeated injections may be considered to achieve good therapeutic effects.

Notably, the rate of adverse events in this study was very low (11.8%), and no major adverse events were reported. Patients benefited from the procedure through improved continence and reduced medication use. When it comes to potential predictors of treatment success, there are currently no reliable prognostic indicators. A careful analysis of the data revealed that children with baseline characteristics such as an older age, lower bladder capacity, and higher detrusor pressure appeared to report higher GRA values. Although these trends were not statistically significant, they should be considered exploratory and hypothesis-generating. Given the small sample size and limited statistical power, the possibility of a type II error (false negative) cannot be excluded. Larger cohorts are required to more reliably identify predictive factors. This may be attributable to the subjective satisfaction derived from noticeable improvements in the patients’ severe baseline conditions. Another limitation of this study is that, for patients receiving repeated BoNT/A injections, changes in clinical or urodynamic outcomes were not tracked over time. While post-treatment urodynamic data were collected after each injection, we did not analyze longitudinal trends such as durability or waning of the therapeutic effects.

A recent meta-analysis demonstrated a statistically significant inverse correlation between the dryness rate and patient age, suggesting a progressive decline in the effectiveness of intravesical BoNT/A injections with increasing age [12]. This finding contrasts with our results, as older children in our cohort reported higher GRA values. One possible explanation is the overall younger age distribution in our study, which may have allowed older children to more accurately articulate their treatment experiences without relying on caregiver interpretation. Variations in study populations, inclusion criteria, and assessment methods may also account for these discrepancies, highlighting that the impact of age on BoNT/A efficacy remains controversial. Furthermore, although the BoNT/A dosing range in our study (5–10 U/kg, up to 200 U) was relatively broad, we did not perform a dose–response analysis due to the small sample size and lack of standardized dosing intervals. In addition, important adjunctive variables—such as the resumption of anticholinergic medications in non-responders, bladder compliance thresholds for initiating clean intermittent catheterization (CIC), and coexisting bowel dysfunction—were not captured in this study, despite their potential influence on patient outcomes and satisfaction. Future prospective studies should incorporate these factors to improve the comprehensiveness and accuracy of predictive modeling.

In addition to clinical effectiveness, several practical issues must be considered when administering intravesical BoNT/A injections in children. These include the requirement for general anesthesia, which adds procedural risk and logistical complexity. Treatment costs and accessibility may also limit availability in some settings, particularly in resource-limited healthcare systems. Furthermore, the need for repeated hospital visits may place a psychological and logistical burden on both the child and their caregivers. These factors are crucial when evaluating BoNT/A as a treatment option and should be further explored in future research.

This study has several limitations that must be acknowledged. First, the small sample size limits the statistical power and generalizability of the findings. As a retrospective study, it is inherently subject to various biases, including the uneven distribution of baseline characteristics. In particular, differences in age, gender, underlying neurological conditions, and methods of bladder management among patients may have influenced the treatment outcomes, introducing potential confounding factors. To mitigate this, we included only patients with complete clinical and urodynamic records across the entire treatment and follow-up period. All outcomes were cross-validated by two independent reviewers. Nonetheless, unmeasured confounding variables may still exist. Conducting examinations in children also presented significant challenges, as many were not well controlled during the procedures, potentially affecting the accuracy of the assessments. For younger children, GRA scores may vary depending on input from different caregivers, introducing subjective variability. Additionally, the outcome assessments were unblinded, as the respondents were aware of the intervention, potentially leading to an overestimation of treatment effects. Future prospective studies should consider incorporating blinded evaluations or objective outcome measures to reduce bias. Errors in estimating bladder capacity may have occurred due to inaccuracies in age calculations. Additionally, the study was conducted at a single institution, which may limit the reproducibility of the results in other clinical settings. These limitations should be taken into account when interpreting the findings and considering their applicability to broader patient populations.

## 4. Conclusions

This study suggests that intravesical BoNT/A injection may be a safe and potentially effective treatment option for pediatric NDO. In our small, single-center, retrospective cohort, the procedure was associated with trends toward improved bladder compliance, increased bladder capacity, and reduced detrusor pressure, although only bladder capacity showed a tendency of improvement. No serious adverse events were observed. These preliminary findings should be interpreted cautiously and validated by larger, prospective studies. For patients who do not respond well to a single injection, repeated injections may be considered with caution, though more robust evidence is needed to support their comparative efficacy. The patient-reported satisfaction appeared favorable, with reductions in both incontinence and medication use. While the trends suggested greater subjective improvement in patients with poorer baseline bladder conditions, no statistically significant predictors of success were identified. These findings may reflect type II error due to the small sample size and should be further investigated in future studies designed with adequate statistical power.

## 5. Materials and Methods

### 5.1. Study Design and Subjects

We retrospectively reviewed the medical records of pediatric patients (those under 18 years old) who received intravesical injections of BoNT/A at Taipei Tzu Chi Hospital between June 2005 and September 2023. If the patient received multiple injections, each injection was recorded separately. We also reviewed records of the patients’ history, clinical symptoms, voiding patterns, and urodynamic investigations. We used each patient’s last follow-up as the main basis for evaluation. All of the considered patients presented with varying bothersome lower urinary tract symptoms, including urinary frequency, urgency, and incontinence, that were refractory to conservative treatment, including oral medications. To ensure data quality, only patients with complete urodynamic data and documented symptoms and response values at baseline and follow-up were included. The records were reviewed independently by two authors (Hsu and Wu), and discrepancies were resolved through consensus. Patients with incomplete data were excluded to avoid imputation bias.

### 5.2. Urodynamic Assessment

All of the considered patients received a complete lower urinary tract evaluation using a video urodynamic study (VUDS) prior to intravesical BoNT/A injection to verify the need for treatment and avoid unnecessary injury. A VUDS was also performed on every patient three months after the first intravesical BoNT/A injections. Patients who saw subsequent improvements had regular follow-ups with non-invasive urodynamic studies using uroflowmetry and bladder ultrasound to determine the residual urine volume. For those who relied on catheterization, a regular voiding diary was used as an alternative. Those who saw limited efficacy and required repeated injections had a VUDS performed prior to each subsequent injection. Among the children who received repeated injections, a VUDS was also performed following the final injection to assess the overall outcome. Patients without a documented VUDS following the first injection were excluded from the analysis. The implementation of the VUDS was based on the recommendations of the standard guidelines of the International Children’s Continence Society (ICCS) [29].

### 5.3. BoNT/A Injection

All participants received a pre-operative evaluation, an intravesical BoNT/A injection, and post-operative care in line with the standard of care at our institution. The injection was performed under general anesthesia during hospitalization. The principle of intravesical injection is 20 trigone-sparing injections using BoNT/A (onabotulinum toxin A, Botox^®^). The specific dose of BoNT/A (5–10 U/kg, up to 200 U) was determined based on the patient’s weight and prior response, per institutional protocol. However, the outcomes were not stratified by dose due to the small sample size and overlapping dosage ranges. Fr. 8/9.8 pediatric cystoscopy was used for small children, while Fr. 16 cystoscopy was used for older pediatric patients to account for the patients’ body sizes and, in turn, to avoid urethral trauma. No additional therapy was provided after the injection until the first clinical follow-up. Patients taking oral medications such as antimuscarinics were instructed to discontinue them after the intravesical BoNT/A injections to prevent overlapping effects and potential side effects.

### 5.4. Follow-Up and Outcome Measures

The first post-injection follow-up was typically scheduled one week after discharge from the hospital to confirm that there were no short-term complications related to the procedure. The next clinical follow-up was then scheduled for three months after the injection, concurrently with the VUDS follow-up. The VUDS parameters were recorded as standard definitions, and the estimated bladder capacity was measured using the following formula: (age in years + 1) × 30 [29,30]. In addition to the VUDS at the three-month post-injection point, the urinary flow rate and residual urine volume were recorded every three months. In patients requiring catheterization, the urine volume obtained from CIC was recorded to assess the functional bladder capacity for follow-up. Oral medications such as antimuscarinics were resumed during the follow-up if there was a poor or unsatisfactory response after intravesical BoNT/A injection.

The patient-reported outcome is another important parameter for measuring the effectiveness of intravesical BoNT/A injection. The children (or their families) were asked to rate the overall changes in their lower urinary tract symptoms. The reported global response assessment (GRA) values were recorded during every clinical follow-up after each injection. The GRA, covering values of −3, −2, −1, 0, 1, 2, and 3, indicates markedly worse, moderately worse, mildly worse, unchanged, mildly improved, moderately improved, and markedly improved symptoms, respectively. A GRA value of ≥2 was defined as successful treatment. All others were defined as treatment failures. Potential side effects associated with the injections were also recorded at each follow-up visit, including urinary tract infection, urine retention, and severe hematuria. The outcome assessment using the GRA was not blinded, as the ratings were obtained directly from the patient or caregiver during follow-up consultations. This introduces a risk of reporting bias, particularly for subjective outcomes. If the numerical part of the GRA score had not been recorded, we would have considered estimating it based on the written notes in the medical records. However, this situation did not occur.

### 5.5. Statistical Analysis

We present the data as medians alongside interquartile ranges (IQRs) to avoid the presence of outliers and skewed raw data. A paired t-test was used to compare the VUDS parameters before and after intravesical BoNT/A injection. We performed subgroup analyses based on the number of injections, dividing patients into single-injection and repeated-injection groups to measure their outcomes separately. In the repeated-injection group, the VUDS parameters after the final injection were used to compare the effectiveness of the entire treatment with that of the single injection. We performed univariate and multivariate logistic regression analyses to determine the predictors of satisfactory therapeutic outcomes. A *p*-value of <0.05 was considered to indicate statistical significance. To account for multiple comparisons across the three primary urodynamic outcomes (compliance, cystometric bladder capacity, and detrusor pressure), Bonferroni correction was applied. The adjusted significance threshold was set at *p* < 0.017 (0.05/3). All statistical analyses were performed using SPSS version 21 (IBM Corp., Armonk, NY, USA).

### 5.6. Research Ethics

This study was conducted in accordance with the principles outlined in the Declaration of Helsinki. Ethical approval was obtained from the Ethics Committee of Taipei Tzu Chi Hospital, Buddhist Tzu Chi Medical Foundation (IRB No.: 12-X-001), and the requirement for informed consent was waived due to the study’s retrospective nature. All data were de-identified to protect patient confidentiality and privacy.

## Figures and Tables

**Table 1:** Baseline demographics, characteristics, and outcomes of the study population (N = 18).

	N (%)
**Age distribution** **(years)**	
0~3	4 (22.2)
3~6	2 (11.1)
6~10	6 (33.3)
10~15	5 (27.8)
>15	1 (5.6)
**Sex**	
Male	14 (77.8)
Female	4 (22.2)
**Underlying disease**	
MMC	14 (77.8)
Pelvic surgery	3 (16.7)
Encephalopathy	1 (5.6)
**Symptoms**	
Frequency	4 (22.2)
Urgency	3 (16.7)
Nocturia	3 (16.7)
Incontinence	9 (50)
Urine retention	13 (72.2)
**Voiding patterns**	
Self-voiding (totally catheter-free)	2 (11.1)
CIC	15 (83.3)
Urethral catheter	1 (5.6)
**Upper urinary tract involvement**	
Hydronephrosis	10 (55.6)
VUR	13 (72.2)
High grade VUR	3 (16.7)
**Adverse events**	
Temporary gross hematuria	0
Unexpected ER visit	0
Urinary tract infection	2 (11.1)
**Outcomes**	
Improving incontinence	8 (44.4)
Decreased oral medications	8 (44.4)
**Satisfaction (GRA = 2, 3)**	14 (77.8)

CIC: clean intermittent catheterization, ER: emergency room, GRA: global response assessment, MMC: myelomeningocele, VUR: vesicoureteral reflux.

**Table 2:** Changes in urodynamic parameters from baseline to post-BoNT/A injection therapy (N = 18).

NDO	Median (IQR)		
	Before Botox	After Botox	*p*-Value
**Compliance**	6.35 (3.06, 11.86)	11.10 (5.66, 18.90)	0.116
**Measured/expected CBC**	0.48 (0.29, 0.67)	0.69 (0.35, 0.90)	0.041
**Detrusor pressure (cmH_2_O)**	24 (5.5, 38.75)	16 (8.5, 34.5)	0.248

CBC: cystometric bladder capacity, NDO: neurogenic detrusor overactivity.

**Table 3:** Changes in urodynamic parameters from baseline to post-BoNT/A injection according to single or repeated injections.

	Single Injection	Repeat Injection	*p*-Value
No. Pts	8	10	
**Compliance**			0.317
Before BoNT/A	9.35 (4.48, 15.64)	3.08 (1.08, 12.24)	
After BoNT/A	18.79 (3.76, 40.57)	9.53 (6.89, 13.13)	
*p*-value	0.345	0.237	
**Measured/expected CBC**			0.908
Before BoNT/A	0.45 (0.34, 0.68)	0.51 (0.26, 0.72)	
After BoNT/A	0.85 (0.39, 0.91)	0.49 (0.30, 0.93)	
*p*-value	0.176	0.161	
**Detrusor pressure (cmH_2_O)**			0.354
Before BoNT/A	25 (15, 38)	16 (3.5, 47.5)	
After BoNT/A	17 (8.25, 33)	16 (8, 41)	
*p*-value	0.116	0.866	

BoNT/A: botulinum toxin-A, CBC: cystometric bladder capacity.

**Table 4:** Baseline demographics and urodynamic parameters in patients with neurogenic bladder: analysis of predictors for postoperative favorable outcomes after intravesical BoNT/A injection.

NDO	GRA 0, 1 (N = 4)	GRA 2, 3 (N = 14)	*p*-Value	*p*-Value Univariate	*p*-Value Multivariate	Odds Ratio
**Age**	5.25 (1.8, 13.13)	8.45 (4.28, 10.08)	0.49	0.711	0.995	1.057 (0.789–1.414)
**Compliance**	6.35 (1.65, X)	6.54 (3.06, 11.52)	0.885	0.975	0.997	0.997 (0.834–1.192)
**Measured/expected CBC**	0.79 (0.51, X)	0.38 (0.26, 0.66)	0.069	0.097	0.994	0.001 (0–3.56)
**Detrusor pressure**	4 (3, X)	24 (12.5, 40)	0.419	0.357	0.999	1.041 (0.95–1.133)

BoNT/A: botulinum toxin-A, CBC: cystometric bladder capacity, GRA: global response assessment, NDO: neurogenic detrusor overactivity.

## Data Availability

The original contributions presented in this study are included in the article. Further inquiries can be directed to the corresponding author(s).
